# Health related quality of life of Iranian children with type 1 diabetes: reliability and validity of the Persian version of the PedsQL™ Generic Core Scales and Diabetes Module

**DOI:** 10.1186/1477-7525-9-104

**Published:** 2011-11-23

**Authors:** Peyman Jafari, Elham Forouzandeh, Zahra Bagheri, Zohreh Karamizadeh, Keivan Shalileh

**Affiliations:** 1 Department of Biostatistics, Shiraz University of Medical Sciences, Shiraz, Iran; 2 Department of Pediatrics, Shiraz University of Medical Sciences, Shiraz, Iran; 3 Medical School, Tehran University of Medical Sciences, Tehran, Iran

**Keywords:** quality of life, diabetes, validation studies, Iran

## Abstract

**Background:**

The aim of this study was to measure health related quality of life (HRQOL) in Iranian children with type 1 diabetes and to test the psychometric properties of the Persian version of the PedsQL™ 4.0 Generic Core Scales and the PedsQL™ 3.0 Diabetes Module.

**Methods:**

Participants were 94 children and adolescents diagnosed with type 1 diabetes for at least 3 months in Shiraz, southern Iran. Convergent, discriminant, and construct validity of the PedsQL™ 4.0 Generic Core Scales and the PedsQL™ 3.0 Diabetes Module were assessed. Moreover, internal consistency was checked by Cronbach's alpha coefficient.

**Results:**

Cronbach's α for the PedsQL™ 4.0 Generic Core Scales and the PedsQL™ 3.0 Diabetes Module was greater than 0.80 both in the child self-report and parent proxy-report. Both generic and disease-specific versions of the PedsQL showed excellent convergent and acceptable discriminant validity except for 'diabetes symptoms' subscale in the child self-report of the disease-specific module. Moreover, Iranian children with diabetes, as compared with other countries, had lower HRQOL scores.

**Conclusions:**

While this study showed that the Persian version of the PedsQL™ 4.0 Generic Core Scales has good psychometric properties in children with type 1 diabetes, the PedsQL™ 3.0 Diabetes Module needs some modifications to be used as a disease-specific quality of life (QOL) measure. Also, more support should be provided for the care of Iranian children with diabetes.

## Background

There is a worldwide increase in the incidence of type 1 diabetes [1]. It affects approximately 1 in every 400-600 children and adolescents [2]. Childhood diabetes adversely affects health related quality of life (HRQOL) of the patients and their families. Diabetes imposes restrictions on physical, emotional and social functioning of children and adolescents. One of the international tools for assessment of HRQOL of children is the Pediatric Quality of Life Inventory (PedsQL). This instrument provides both child self-report and parent proxy-report. The generic form of this questionnaire has been widely used in many countries and it has shown acceptable psychometric properties among children and their parents [3-9]. Moreover, the PedsQL™ 4.0 Generic Core Scales and the PedsQL™ 3.0 Diabetes Module have been used to measure the quality of life (QOL) in children with diabetes, having good reliability and validity [10-14]. Although, the feasibility of the Persian version of the PedsQL™ 4.0 Generic Core Scales has recently been approved among the general population and in children with attention deficit/hyperactivity disorder (ADHD) [15,16], the psychometric properties of this questionnaire are still unknown in Iranian children with other chronic diseases. Measuring quality of life in Iranian children and adolescents with diabetes and assessment of reliability and validity of the Persian version of the PedsQL™ 4.0 Generic Core Scales and the PedsQL™ 3.0 Diabetes Module are the main goals of this study.

## Methods

### Participants and instruments

Ninety-four children and adolescents, aged 8-18, diagnosed with type 1 diabetes for at least 3 months and their parents (95% mothers) referring to tertiary care clinics of Shiraz University of Medical Sciences, Iran, were enrolled in the study. They completed the same Persian version of the PedsQL™ 4.0 Generic Core Scales, which was previously translated and validated by two studies in Iran [15,16]. They also filled in the translated Persian version of the PedsQL™ 3.0 Diabetes Module. The control subjects were 200 school children, aged 8-18, and their parents who completed the PedsQL™ 4.0 Generic Core Scales. They were randomly selected in a two-stage cluster random sampling from the four educational districts of Shiraz, Southern Iran. The case and control groups were matched for gender. The participants' characteristics are shown in Table 1.

**Table 1:** The characteristics of the case and control groups

	Diabetic children	Healthy children
		
	No (%)	No (%)
Sex		
Male	41(43.6)	94(47)
Female	53(56.4)	106(53)
		
Age group (year)		
8-12	61(64.9)	63(31.5)
13-18	33(35.1)	137(68.5)
Mean ± SD	10.94 ± 3.32	14.24 ± 2.29
		
Insulin injection (per day)		
1	6(6.4)	-
2	85(90.4)	-
3	3(3.2)	-
		
Length of treatment (Mean ± SD)		
Male	22.05 ± 22.39	-
Female	27.53 ± 19.35	-

The PedsQL™ 4.0 Generic Core Scales consisted of 23 items divided into four subscales including physical, emotional, social, and school functionings with 8, 5, 5 and 5 items, respectively. The questionnaires took approximately 10 minutes to be completed. Likert response scale with five categories was used, ranging from never a problem (0) to almost always a problem (4). All the subscales were transformed to a 0-100 score so that higher scores represented better QOL. One of the authors (E.F) was responsible for completing the questionnaire for children by face-to-face interview and she was also available to clarify the possible questions of the parents about the instrument. Permission to use the translated Persian version of the PedsQL™ 3.0 Diabetes Module was obtained from the developer of the questionnaire by the first author. The PedsQL™ 3.0 Diabetes Module used in this study was translated into Persian from the original questionnaire using the linguistic validation of the PedsQL™ protocol. This disease-specific module includes 28 questions to assess 5 subscales of HRQOL, which include diabetes symptoms (11 items), treatments barriers (4 items), treatment adherence (7 items), worry (3 items) and communications (3 items). Moreover, its Likert scale and subscale scores were similar to the PedsQL™ 4.0 Generic Core Scales. The children and their parents were informed about the study and its aim and gave signed informed consent.

### Statistical analysis

The reliability of the QOL subscales was tested using the Cronbach's alpha coefficient. Internal consistency was considered satisfactory if the coefficient was at least 0.7. The exploratory factor analysis with Varimax rotation was used to determine the construct validity of the PedsQL™ 4.0 Generic Core Scales. Convergent and discriminant validity was checked using Spearman correlation. The value of a correlation coefficient of greater than 0.40 between an item and its own scale is regarded as an adequate evidence of convergent validity. Discriminant validity is supported whenever a correlation between an item and its hypothesised scale is higher than its correlation with the other scales. A scaling success is counted if the item to own-scale correlation is significantly higher than the correlations of the item to other scale [17]. Analysis of covariance with adjustment for age was used to compare HRQOL between Iranian children with and without diabetes.

## Results

Table 2 displays the Cronbach's alpha coefficients, means and SDs of the PedsQL™ 4.0 Generic Core Scales for children with and without diabetes and for the PedsQL™ 3.0 Diabetes Module. It shows that all of the domains for both of the versions of the PedsQL have sufficient reliability which is greater than 0.7 except for 'treatment barriers' in children and parents in Diabetes Module. Moreover, for all domains except for social functioning, diabetic children reported their HRQOL lower than children without diabetes. The results of the convergent and discriminant validity for the PedsQL™ 4.0 Generic Core Scales and the PedsQL™ 3.0 Diabetes Module are presented in Table 3. These findings show that the scaling success rates for convergent validity is 100% for all domains except for physical health in generic form and diabetes symptoms in Diabetes Module. The success rate for item discriminant validity of the PedsQL™ 4.0 is 88% (61/69) and 87% (60/69) for children and parents, respectively. The success rate for item discriminant validity of the PedsQL™ 3.0 Diabetes Module is 89% (100/112) and 78% (87/112) for children and parents, respectively. The result of the factor analysis with Varimax rotation to test the construct validity of the PedsQL™ 4.0 Generic Core Scales is presented in Table 4. The proportions of variance explained by the first four factors are 56% and 58% for self- and proxy-report, respectively. In child self-report, the items of 'Hard to take a bath or shower' and 'Hurt or ache' in physical health, 'Trouble sleeping' in emotional functioning, and 'Doing things other peers do' and 'Hard to keep up when play with others' in social functioning have correlations below 0.4. Moreover, in parent proxy-report, the items of 'Hurt or ache' and 'Low energy' in physical health, 'Doing things other peers do' and 'Hard to keep up when play with others' in social functioning and 'Miss school - not well' in school functioning have a weak correlation with their own domains.

**Table 2:** Cronbach's alpha for the generic and diabetic module in Iranian children with diabetes and score subscales for diabetic and school children

		Diabetic children			School children				
Scale	No.items						F	d.f	p-value
		n	mean ± SD	α	n	mean ± SD			
**PedsQL™ 4.0 Generic**									
**Core Scales**									
*Child self-report*									
Total score	23	94	67.98 ± 14.03	0.87	200	78.21+13.23	33.33	(1,292)	< 0.001
Physical health	8	94	69.18 ± 17.05	0.77	200	80.06+15.02	29.34	(1,292)	< 0.001
Emotional functioning	5	94	59.84 ± 20.41	0.71	200	69.17+19.60	13.93	(1,292)	< 0.001
Social functioning	5	94	76.78 ± 18.19	0.73	200	78.62+18.25	1.69	(1,292)	0.194
School functioning	5	94	65.79 ± 18.23	0.73	200	83.90+16.59	52.54	(1,292)	< 0.001
Psychosocial health	15	94	67.34 ± 14.72	0.82	200	77.23+14.36	26.15	(1,292)	< 0.001
*Parent proxy-report*									
Total score	23	94	67.03 ± 12.83	0.83	200	74.79+16.22	14.91	(1,292)	< 0.001
Physical health	8	94	67.42 ± 16.50	0.73	200	76.00+20.11	12.03	(1,292)	0.001
Emotional functioning	5	94	58.63 ± 18.48	0.73	200	68.02+21.09	16.37	(1,292)	< 0.001
Social functioning	5	94	75.16 ± 18.63	0.71	200	81.30+20.90	2.99	(1,292)	0.085
School functioning	5	94	66.65 ± 19.24	0.72	200	73.12+20.68	5.61	(1,292)	0.019
Psychosocial health	15	94	66.81 ± 13.81	0.79	200	74.15+16.59	12.13	(1,292)	0.001
**PedsQL™ 3.0 Diabetic**									
**Module**									
*Child self-report*									
Diabetes symptoms	11	94	59.21 ± 14.46	0.74	-	-	-	-	-
Treatment barriers	4	94	58.38 ± 21.51	0.67	-	-	-	-	-
Treatment adherence	7	94	60.18 ± 18.12	0.78	-	-	-	-	-
Worry	3	94	56.47 ± 23.35	0.71	-	-	-	-	-
Communication	3	94	61.08 ± 27.34	0.76	-	-	-	-	-
*Parent proxy-report*									
Diabetes symptoms	11	94	58.57 ± 13.62	0.73	-	-	-	-	-
Treatment barriers	4	94	53.53 ± 21.03	0.68	-	-	-	-	-
Treatment adherence	7	94	57.60 ± 15.99	0.71	-	-	-	-	-
Worry	3	94	56.91 ± 23.01	0.72	-	-	-	-	-
Communication	3	94	60.99 ± 25.69	0.70	-	-	-	-	-

*P-value is adjusted based on age

**Table 3:** Item scaling tests: convergent and discriminant validity for the PedsQL™ 4.0 Generic Core Scales and the PedsQL™ 3.0 Diabetes Module subscales

		Convergent validity^a^		Discriminant validity^b^	
			
Scale	**No**. items	Range of correlation	Scaling success (percent)	Range of correlation	Scaling success (percent)
**PedsQL™ 4.0 Generic Core Scales**					
*Child self-report*					
Physical health	8	0.52-0.72	8/8 (100)	0.05-0.47	20/24 (83)
Emotional functioning	5	0.48-0.75	5/5 (100)	0.07-0.46	12/15 (80)
Social functioning	5	0.65-0.73	5/5 (100)	0.18-0.54	13/15 (87)
School functioning	5	0.51-0.79	5/5 (100)	0.11-0.34	15/15 (100)
*Parent proxy-report*					
Physical health	8	0.36-0.72	7/8 (87.50)	0.006-0.46	19/24 (79)
Emotional functioning	5	0.60-0.80	5/5 (100)	0.004-0.37	15/15 (100)
Social functioning	5	0.51-0.82	5/5 (100)	0.07-0.47	14/15 (93)
School functioning	5	0.46-0.81	5/5 (100)	0.005-0.47	13/15 (87)
					
**PedsQL™ 3.0 Diabetic Module**					
*Child self-report*					
Diabetes symptoms	11	0.37-0.66	10/11 (90.91)	0.001-0.42	28/44 (64)
Treatment barriers	4	0.63-0.75	4/4 (100)	0.26-0.74	14/16 (88)
Treatment adherence	7	0.58-0.82	7/7 (100)	0.16-0.62	21/28 (75)
Worry	3	0.70-0.85	3/3 (100)	0.22-0.61	12/12 (100)
Communications	3	0.76-0.87	3/3 (100)	0.25-0.50	12/12 (100)
*Parent proxy-report*					
Diabetes symptoms	11	0.43-0.65	11/11 (100)	0.002-0.42	39/44 (89)
Treatment barriers	4	0.58-0.83	4/4 (100)	0.19-0.57	14/16 (88)
Treatment adherence	7	0.54-0.71	7/7 (100)	0.01-0.54	23/28 (82)
Worry	3	0.75-0.84	3/3 (100)	0.20-0.50	12/12 (100)
Communications	3	0.69-0.86	3/3 (100)	0.13-0.49	12/12 (100)

a. Number of correlation between items and hypothesized scale corrected for overlap ≥ 0.4/total number of convergent validity tests.

b. Number of convergent correlations significantly higher than discriminant correlations/Total number of correlations.

**Table 4:** Factor loadings (rotated)^1 ^of four factor solution of the PedsQL™ 4.0 Generic Core Scales

Scale	Child self-report				Parent proxy-report			
		
	F1	F2	F3	F4	F1	F2	F3	F4
**Physical health**								
1. Hard to walk more than a block	**0.73**	0.14	0.12	0.02	**0.79**	-0.03	-0.02	0.04
2. Hard to run	**0.70**	-0.006	0.25	0.01	**0.86**	0.01	0.00	-0.06
3. Hard to do sports or exercises	**0.71**	-0.11	0.42	-0.004	**0.73**	-0.07	0.25	-0.004
4. Hard to lift something heavy	**0.63**	0.06	0.03	0.25	**0.51**	0.26	0.21	0.03
5. Hard to take a bath or shower	**0.25**	0.17	0.57	0.08	**0.40**	0.00	0.67	-0.11
6. Hard to do chores around house	**0.44**	0.23	0.03	0.24	**0.55**	0.39	0.10	0.13
7. Hurt or ache	**0.24**	0.45	0.24	0.26	**0.13**	0.62	0.40	0.02
8. Low energy	**0.41**	0.39	0.19	-0.12	**0.20**	0.51	0.10	0.06
**Emotional functioning**								
1. Feel afraid or scared	0.06	**0.68**	0.28	-0.004	0.04	**0.76**	0.10	-0.03
2. Feel sad or blue	0.15	**0.71**	0.06	-0.034	0.16	**0.74**	-0.29	0.08
3. Feel angry	0.17	**0.58**	0.12	0.13	0.01	**0.44**	-0.36	0.28
4. Trouble sleeping	0.16	**0.25**	0.32	0.42	0.04	**0.54**	0.05	0.16
5. Worry about what will happen	0.01	**0.78**	-0.07	0.14	0.10	**0.74**	0.27	-0.08
**Social functioning**								
1. Trouble getting along with peers	0.13	0.26	**0.71**	0.25	0.12	0.07	**0.70**	0.29
2. Other kids not wanting to be friends	0.05	0.48	**0.48**	0.37	0.20	0.25	**0.73**	0.24
3. Teased	0.18	0.16	**0.70**	0.12	0.10	0.20	**0.69**	0.28
4. Doing things other peers do	0.58	0.30	**-0.03**	0.22	0.32	0.15	**0.04**	0.28
5. Hard to keep up when play with others	0.55	0.28	**0.00**	0.14	0.46	0.24	**-0.06**	0.39
**School functioning**								
1. Hard to concentrate	-0.01	-0.02	0.26	**0.78**	0.17	0.05	0.42	**0.70**
2. Forget things	0.08	0.13	0.10	**0.74**	0.02	0.24	0.15	**0.69**
3. Trouble keeping up with schoolwork	0.14	-0.11	0.30	**0.69**	0.07	-0.06	0.37	**0.63**
4. Miss school - not well	0.23	0.13	-0.20	**0.58**	0.35	-0.06	0.23	**0.28**
5. Miss school - doctor appointment	0.22	0.19	-0.47	**0.48**	0.14	-0.07	-0.05	**0.73**

1: Varimax rotation.

F1: Physical functioning, F2: Emotional functioning, F3: Social functioning, F4: School functioning.

Items belonging to the postulated scales are shown by bold numbers.

Factor loadings under 0.4 have been underlined.

## Discussion

This study indicates that the Persian version of the PedsQL™ 4.0 Generic Core Scales is a reliable instrument in Iranian children with type 1 diabetes, with excellent convergent and good discriminant validity. Factor analysis provides evidence that the Persian version of the generic form encompasses four underlying constructs, namely, physical, emotional, social, and school functioning. This is in agreement with the findings of a previous study on the psychometric properties of this questionnaire in Iranian children with attention deficit/hyperactivity disorder (ADHD) [16]. However, our findings were not identical to those of the English version [11]; e.g., 'Hard to take a bath or shower' and 'Hurt or ache' in child self-report were strongly correlated with emotional functioning rather than physical functioning. These findings reveal that phrases such as 'Hurt or ache' do not convey similar meaning in Iranian and American children: In Iranians, 'Hurt or ache' frequently referred more to mood states rather than somatic responses. Similar results were obtained for parents. Also, two items of the social functioning, namely, 'Doing things other peers do' and 'Hard to keep up when play with others', both in self- and proxy-reports, are clearly associated with physical rather than social functioning. It seems that these items were considered as a physical limitation which prevented children from doing things that their peers are able to do [15]. Moreover, all emotional and school functioning items had a clear factor loading except for item 4 'Trouble sleeping' in emotional functioning and 'Miss school - not well' in school functioning for child self-report and parent proxy-report, respectively. Although the Persian version of the PedsQL™ 3.0 Diabetes Module has good reliability and convergent validity, caution is warranted when interpreting the discriminant validity of the 'diabetes symptoms' subscale in children.

In this study, construct validity of the diabetes module has not been assessed because the sample size (94 patients) is rather small to conduct a stable factor analysis. Despite this limitation, we reported the results of the factor structure of the PedsQL™ 4.0 Generic Core Scales in order to compare our findings with those from previous studies [15,16].

In comparison with Greek [9,10], American [11,12], and Dutch [14] children, Iranian children and adolescents with type 1 diabetes reported lower HRQOL scores in all domains, according to self- and proxy-reports using the PedsQL™ 4.0 Generic Core Scales and the PedsQL™ 3.0 Diabetes Module. While Greek children rated their HRQOL significantly higher than their parents, there was no statistically-significant difference between child self-report and parent proxy-report in Iranians. The lower HRQOL in Iranian diabetic children could be ascribed to inadequate health care services or lack of knowledge of parents about the needs of their children.

Moreover, as compared with Iranian children with ADHD, children in our study had significantly higher HRQOL scores in all domains [16]. However, the quality of life of Iranian children with diabetes is lower than that of the school children which is very similar to the findings in previous studies [1,10-12].

This study has a number of potential limitations. First the school children were significantly older than the diabetic children. Therefore, subscale scores were compared between the two groups using age of subject as covariate in an analysis of covariance design (ANCOVA). We found no correlation between age and subscale scores and hence ANCOVA could not show the effect of age and did not change the principal findings. Moreover, due to the small sample size, the results of convergent, discriminant and internal consistency were not reported by age group. Because of this, we cannot be sure of the external validity of self- and proxy-reports for the children of age 13-18 years.

In this study, the compliance rate was 82% (94/115). The main reasons for not completing the questionnaires were generally the parental illiteracy, busy clinic, and the relatively large number of items in the generic and disease-specific instruments. Children and their parents felt it was inconvenient and completing it took too long. Also, they felt it was a violation of their privacy.

## Conclusions

In conclusion, although this study showed that the Persian version of the PedsQL™ 4.0 Generic Core Scales and the PedsQL™ 3.0 Diabetes Module have good psychometric properties in children with type 1 diabetes, diabetes module needs some modifications to be used as a disease-specific QOL measure. Moreover, lower HRQOL in Iranian children with diabetes indicates that youth with diabetes in Iran require intensive programs to increase their HRQOL, and more supportive resources should be allocated.

## List of abbreviations

HRQOL: Health Related Quality of Life; QOL: Quality of Life.

## Competing interests

The authors declare that they have no competing interests.

## Authors' contributions

PJ analyzed and wrote the manuscript and researched the data, EF researched the data, ZB wrote the manuscript and analyzed the data, KSh researched the data and edited the manuscript, ZK researched the data. All authors read and approved the final manuscript.
